# Effects of Endurance Training on Antioxidant and Hormonal Status in Peripheral Blood of Young Healthy Men

**DOI:** 10.3390/life14080921

**Published:** 2024-07-24

**Authors:** Stanimir Stojiljković, Ljubica Gavrilović, Snežana Pejić, Snežana B. Pajović, Marija Macura, Dragan Nikolić, Saša Bubanj, Vesna Stojiljković

**Affiliations:** 1 Faculty of Sport and Physical Education, University of Belgrade, 11000 Belgrade, Serbia; stanimir.stojiljkovic@fsfv.bg.ac.rs (S.S.); marija.macura@fsfv.bg.ac.rs (M.M.); 2 Department of Molecular Biology and Endocrinology, “Vinča” Institute of Nuclear Sciences, National Institute of the Republic of Serbia, University of Belgrade, 11000 Belgrade, Serbia; gljubica@vin.bg.ac.rs (L.G.); snezana@vin.bg.ac.rs (S.P.); pajovic@vin.bg.ac.rs (S.B.P.); 3 School of Medicine, University of Belgrade, 11000 Belgrade, Serbia; dragannikolic8@yahoo.com; 4 Clinic for Endocrinology, Diabetes and Metabolic Diseases (Laboratory for Cells Culture), Clinical Centre of Serbia, 11000 Belgrade, Serbia; 5 Faculty of Sport and Physical Education, University of Niš, 18000 Niš, Serbia; bubanjsale@gmail.com

**Keywords:** endurance training, running, antioxidant system, catecholamines, stress

## Abstract

(1) Background: Physical activity may cause an imbalance in the major functions of the human body. This study aimed to investigate the effects of endurance running training on the parameters of the antioxidant defense system (SOD, CAT, GPx, GR, GSH), LPO (malondialdehyde, MDA), and stress hormones (A, NA) in young healthy, previously untrained men. (2) Methods: The training program was as follows: 8 weeks of running, three times per week; the duration of a single session was 30–70 min, the intensity was twice a week in the so-called extensive endurance zone, and once a week in the anaerobic threshold zone. Blood samples were collected from the subjects, before and after the running program. (3) Results: The training program resulted in a significant increase in maximal oxygen consumption (*p* < 0.001). The activities of SOD, GPx, and GR also increased significantly (*p* < 0.05, *p* < 0.01, and *p* < 0.05, respectively), while CAT activity and GSH and MDA concentrations remained unchanged. The concentration of A decreased (*p* < 0.05), while the NA concentration increased significantly (*p* < 0.05). SOD, GPx, GR, and NA positively correlated with VO_2_max (*p* < 0.05, *p* < 0.001, *p* < 0.01, *p* < 0.05, respectively), while a negative correlation was detected between A and VO_2_max (*p* < 0.05). (4) Conclusions: These results indicate that there is no persistent oxidative stress in response to the applied 8-week running program, probably due to exercise-induced protective alterations in the antioxidant defense system. Furthermore, adaptations occurred at the hormonal level, making the organism more ready for a new challenge.

## 1. Introduction

Regular physical activity has been found to decrease the overall level of stress and ameliorate and prevent stress-related diseases [1,2,3,4,5]. Stress is considered a state of threatened homeostasis/balance, with biological consequences such as altered neurohormonal, inflammatory, and autonomic activity that can cause a variety of diseases, including cardiovascular diseases [6], obesity [7], diabetes [8], depression [9], etc. Various stressors (psychological, physical, immunological, metabolic, or environmental) elicit a stress response leading to physiological, behavioral, and psychological adjustments that increase the chance of an organism persisting in stress conditions [10]. Two crucial components of the autoregulatory stress response are the hypothalamic–pituitary–adrenal (HPA) axis and the sympathoadrenal medullary (SAM) system. These two systems work together to maintain homeostasis in stress conditions. Adaptation to the existing environment and anticipation of future challenges are positive side effects of the stress response [11]. However, if the challenging conditions persist, the response to chronic stress may provoke the maladaptation of neuroendocrine regulatory mechanisms and as a consequence, induce turbulent blood flow, hypertension, inflammation, oxidative stress, atherosclerosis, impaired memory, etc. [12,13]. The activation of the HPA axis and the SAM system results in the release of hormones such as adrenocorticotropic hormone, cortisol, prolactin, adrenaline, and noradrenaline, with concomitant withdrawal of the parasympathetic nervous system.

Although stress is recognized as one of the most important risk factors for many diseases, it is still not a major focus of preventive strategies [14]. Physical activity is one of the most important nonpharmacologic therapies for stress-induced diseases, along with relaxation techniques, meditation, mental training, etc. Previous studies have shown that physical activity contributes to reduced reactivity, not only to acute physical stressors [15,16] and life stressors [17,18], but also to psychosocial stressors, with elite athletes showing lower adrenocortical, autonomic, and psychological responses to stress compared to those of untrained men [19].

Physical activity is a stress in itself, and it causes an imbalance in the major functions of the body of the athlete. To restore and maintain the balance of these functional systems, the nervous and endocrine systems generate very powerful mediators such as adrenaline (A) and noradrenaline (NA), whose concentrations alter depending on the duration, intensity, and type of exercise [20].

Physical activity is followed by increased oxygen consumption, and as a consequence, oxidative stress may occur. Oxidative stress is a phenomenon which represents an imbalance between the production of reactive oxygen species (ROS) and the ability of biological defense systems to scavenge them and to repair the resulting damage. While excessive oxidant pressure causes damage to biomolecules, maintenance of a physiological level of oxidant challenge is essential for governing life processes through redox signaling [21]. To counteract the oxidant challenge, the cells have developed antioxidant (AO) defense systems, which include enzymatic (superoxide dismutase—SOD, catalase—CAT, glutathione peroxidase—GPx, glutathione reductase—GR) and nonenzymatic (vitamins A, C, E, glutathione (GSH), retinol, bilirubin, coenzyme Q10) antioxidants. The oxidation of membrane lipids, known as lipid peroxidation (LPO), is one of the first consequences of oxidative stress. It leads to a loss of membrane fluidity and a disruption of its integrity and function [22]. Chain-breaking antioxidants can prevent the damage provoked by LPO, but prolonged LPO may deplete cellular antioxidants and reduce the cell’s AO capacity [23].

The literature data regarding the effects of exercise on oxidative stress and AO defense are inconsistent. Many studies have shown that physical activity induces not only oxidative stress [24,25,26], but also adaptations in AO defense systems [27,28,29]. Similar to changes in catecholamine concentrations, the degree of oxidative damage caused by physical activity depends on the type, intensity, volume, and duration of exercise [30]. The conclusion of a systematic review and meta-analysis by de Sousa et al. [31] revealed that regardless of intensity, volume, and type of exercise, the AO indicators tended to increase, and pro-oxidant indicators tended to decrease after training. On the other hand, the result of another systematic review suggests that exercise does not elicit a response to specific biomarkers of oxidative stress [32].

The main question in the present study was whether the 8-week endurance running training could influence the AO defense system and stress hormones in young, healthy, previously untrained men. We presumed that the applied training program would elicit physiological adaptations that may improve exercise capacity, as well as the stress response. To assess this issue, we compared the initial (before the training program) and the final (after the program) values of the parameters of the AO defense system (SOD, CAT, GPx, GR, GSH), LPO (MDA) and stress hormones (A and NA) in the blood of 42 subjects who participated in the program. 

## 2. Materials and Methods

### 2.1. Subjects

**Figure 1:**
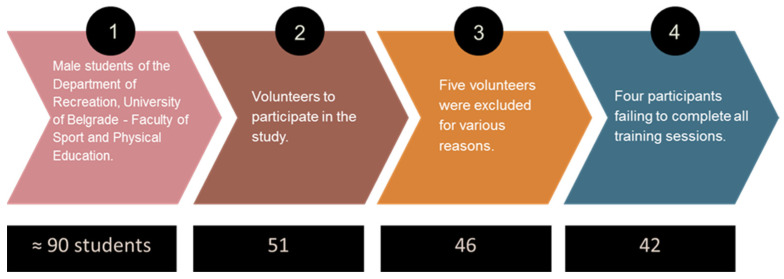
The process of selecting sample subjects.

A total of 51 male students (out of a total of about 90) from the Department of Recreation, University of Belgrade—Faculty of Sport and Physical Education, volunteered to participate in the study. To be included in the study, students had to be healthy and free of musculoskeletal injuries or other conditions that could hinder their participation. The criteria for non-inclusion in the study (exclusion criteria) were: history of chronic diseases; history of acute illness, <3 months before the survey; injury to the locomotor system, especially the legs, <3 months before the study; smoking (smoker or former smoker); body mass index (BMI) > 30 kg/m^2^; blood pressure at rest higher than 160/100 mm Hg; use of drugs that affect cardiovascular capacity; regular, planned and programmed exercise more than three times a week. Five volunteers were excluded for various reasons (two were smokers, two ran regularly 3–5 times a week and ran 5 km races and half marathons, and one had a knee injury one month before the start of the study). A total of 46 male students, with no previous running training experience, participated in this study. Four participants were excluded, due to failing to complete all training sessions. The final sample included 42 healthy volunteers, physically active physical education male students (age 21.7 ± 2.3 years; height 179.9 ± 7.5 cm; weight 76.6 ± 8.4 kg), who successfully completed the experimental protocol. The recruitment, selection, and final number of participants are shown in Figure 1.

All participants were informed of the study procedures, benefits, and potential risks and provided written informed consent. During the experimental period, the subjects were advised to stick to the usual diet and to avoid the use of supplementation or medications. The study was approved by the Ethical Committee of the University of Belgrade—Faculty of Sport and Physical Education (approval number 02-2957/23-2). All procedures were conducted following the standards established by the Declaration of Helsinki and its later amendments.

### 2.2. Incremental Running Test

Before and after the 8-week running program, the participants completed a graded exercise test on the treadmill to measure the first ventilatory threshold (VT1), the second ventilatory threshold (VT2), and the maximal oxygen consumption (VO_2_max). 

In a three-zone model of the exercise [33], the training zones (low, medium, and high intensity) are separated by thresholds, which can be either determined through the ventilatory exchange–ventilatory thresholds (VT1, VT2) [34], or the lactate concentration–lactate thresholds (LT1, LT2) [35].

VT1 is a point where lactate begins to accumulate in the blood and can be observed in an exerciser, whose breathing rate begins to increase and from that point, he/she cannot speak normally, but can still string a few words together. VT2 is a marker of higher exercise intensity, when lactate builds up in the bloodstream faster than the body can remove it; the exerciser breathes very heavily and can no longer speak. VT1 is also called the aerobic threshold (AeT)—usually estimated to be around 60–70% of the maximal heart rate—(HRmax), and VT2 is often called the anaerobic threshold (AnT)—usually estimated to be around 85–90% of the HRmax [36]. 

VO_2_max is achieved during large muscle mass exercise and represents the integrative ability of the heart to generate a high cardiac output, total body hemoglobin, high muscle blood flow, and muscle oxygen extraction, and in some cases the ability of the lungs to oxygenate the blood [37]. In other words, VO_2_max is the maximal ability of the body to take in, transport, and utilize oxygen and is a good measure of a person’s cardiorespiratory fitness. 

Subjects were advised to avoid alcohol and any form of physical activity for a minimum of 48 h prior to testing. A graded exercise test was performed on the same day that blood samples were taken from the subjects. Blood samples were taken earlier in the morning, and a graded exercise test was performed about an hour later. The participants were instructed to eat only a light snack and not to consume caffeine after blood sampling and before coming to the test. The participants started with a standardized 5 min warm-up (3 min walking at 2 km/h, and 2 min walking at 6 km/h), before completing a maximal incremental running test with an initial speed of 8 km/h, which increased by 2 km/h every 2 min, until VO_2_max was reached. Gas exchange data were collected breath-by-breath (Erich Jaeger GmbH, Friedberg, Germany).

The VT1 was determined according to three validated methods to determine VT1 from incremental exercise test data: (1) the modified V-slope method; (2) the ventilatory equivalent method (VE/VO_2_ method); and (3) the end-tidal O_2_ pressure method (PetO_2_). The VT2 was determined using the ventilation/carbon dioxide production (VE/VCO_2_) plot at the point where VE increases out of proportion to the VCO_2_ [35]. 

VO_2_max (mL·kg^−1^·min^−1^) was calculated using the highest VO_2_ measurement average in 30 s. The presence of the plateau of VO_2_ was investigated and confirmed when the VO_2_ remained stable (less than 150 mL O_2_ variation) for at least 30 s, despite the workload increase [38]. In case the plateau was not clearly expressed, two more criteria were observed in combination: (1) maximum HR at a value ≥ 90% of the theoretical maximum HR; (2) respiratory exchange ratio (RER) ≥ 1.10 [39].

### 2.3. Endurance Running Program

The training program aimed to improve aerobic endurance, which is reflected in an increase in VO_2_max, as well as oxygen consumption at VT1 and VT2. The endurance training program was as follows: 8 weeks of running, three times per week; the duration of a single training session was 30–70 min in different intensity zones, based on the results of a graded exercise test on the treadmill. The duration of each training session was the same for all subjects, and the intensity was expressed as running pace (min: s/km) and individually determined based on the running speed at VT1 and VT2, achieved in the incremental running test. 

To improve performance in recreational runners, existing evidence recommends incorporating one to two high-intensity interval training sessions per week, along with several sessions of moderate- and low-intensity continuous submaximal running, into the training regimen [40,41].

The subjects in the present study ran longer training sessions at an intensity just below VT1 level (about 5 to 10 s per kilometer slower than VT1 pace) twice a week (Monday and Friday). That running pace corresponds to the zone of “extensive endurance” and is often described as a long slow distance—LSD—or long run. It is zone 2 in the common classification of five exercise intensity training zones, as recommended by renowned authors [42,43]. It is the zone where the different categories of sub-elite marathon runners run 62.6–67.5% of the weekly distance, the heart rate in zone 2 is in the range of 73–80% of the maximum heart rate, the training sessions last 1–3 h, and the rating of perceived exertion is 3–4 (light) on the Borg scale of 1–10 [44]. 

Once a week (Wednesday), the subjects ran shorter distances at an intensity just below VT2 level (about 5 to 10 s per kilometer slower than VT2 pace). That running pace corresponds to zone 4 in the common classification of five exercise-intensity training zones [42,43]. This zone is often called “anaerobic threshold” or “threshold training”; the heart rate in zone 4 is in the range of 87–92% of the maximum heart rate, and the training sessions last 30–60 min (divided into 4 to 10 intervals of running at the intensity of the anaerobic threshold lasting from 3 to 15 min, with breaks of light jogging lasting 1 to 5 min), and the rating of perceived exertion is 7–8 (hard) on the Borg scale of 1–10 [44,45]. 

Warming up at the beginning and cooling down at the end of training was an integral part of every training session, especially before and after training in zone 4. All training sessions were performed on the 400 m track. The subjects used a stopwatch to control their running pace at each training session. 

### 2.4. Sample Preparation

Blood samples were also collected from the subjects before and after the 8-week running program, on the days when the participants completed the graded exercise test. The participants were asked not to eat any food for 12 h before blood sampling. Blood samples were collected between 8 and 9 a.m. in tubes containing lithium heparin as an anticoagulant and divided in aliquots for different assays. For the SOD assay, the blood was centrifuged at 2500× *g*, 4 °C, for 5 min (Eppendorf centrifuge 5417, Eppendorf AG, Hamburg, Germany). The supernatant was discarded, and the blood cells were washed three times in cold saline and hemolyzed in four volumes of ice-cold demineralized ultra-pure water (MilliQ reagent grade water system, Millipore Corp., Bedford, MA, USA). Then, ethanol-chloroform (1/0.6 vol/vol) extraction was performed to eliminate hemoglobin interference. The extracts were stored at −70 °C until analysis. For the measurement of CAT, GPx, and GR activities, as well as for the determination of the concentrations of MDA and catecholamines, the blood was centrifuged at 1000× *g*, 4 °C for 15 min. The supernatants (plasma) were stored at −70 °C for MDA, A, and NA assays, while the buffy coat was discarded. The blood cells were washed in cold saline three times and then hemolyzed in 20 volumes of ice-cold demineralized ultra-pure water (MilliQ reagent grade water system, Millipore Corp., Bedford, MA, USA). To separate the blood cell stroma, the lysates were centrifuged at 8500× *g*, 4 °C for 10 min, and supernatants were stored at −70 °C for CAT, GPx, and GR assays. For the GSH assay, the whole blood was stored at −70 °C until analysis.

### 2.5. Hemoglobin Assay

The hemoglobin (Hb) concentration is measured using the cyanmethemoglobin method. Hb is released from erythrocytes by osmotic lysis and then oxidized in the presence of potassium cyanide to form a stable complex compound cyanmethemoglobin, whose absorbance is measured at 546 nm. The concentration of Hb is directly proportional to A_546_.

### 2.6. AO Enzyme Activity Assays

All AO enzyme activities were determined spectrophotometrically, using a Perkin Elmer Lambda 25 Spectrophotometer (Perkin Elmer Instruments, Norwalk, CT, USA). The SOD, GPx, and GR activities were measured using appropriate assay kits (Bioxytech^®^ SOD-525TM, Bioxytech^®^ GPx-340TM, Bioxytech^®^ GR-340TM, respectively) obtained from Oxis International Inc., Portland, OR, USA. All procedures were performed according to the manufacturer’s instructions, as previously described [46]. CAT activity was determined according to the method of Beutler [47]. The specific enzyme activity of SOD was expressed in units per gram of hemoglobin (U/gHb); CAT activity was expressed as kilounits per gram of hemoglobin (kU/gHb), while GPx and GR activities were expressed in milliunits per gram of hemoglobin (mU/gHb).

### 2.7. GSH and MDA Assays

The concentrations of GSH and MDA were determined spectrophotometrically, using a Perkin Elmer Lambda 25 Spectrophotometer (Perkin Elmer Instruments, Norwalk, CT, USA) and corresponding assay kits by Oxis International Inc., Portland, OR, USA (Bioxytech^®^ GSH-420TM and Bioxytech^®^ MDA-586TM, respectively), following the manufacturer’s recommendations, as previously described [46]. The GSH concentration was expressed as micromoles per liter of blood, and the MDA concentration as micromoles per liter of plasma (μmol/L).

### 2.8. A and NA Assays

For assessment of the A and NA concentrations, 3-CAT Research ELISA kits (Labor Diagnostica Nord, Nordhorn, Germany) were used, and the manufacturer’s protocols were followed. The absorbances were measured at 450 nm using a Stat Fax 2100 (Awareness Technology Inc., Palm City, FL, USA) microplate reader. The values were expressed as picograms per milliliter of plasma (pg/mL).

### 2.9. Statistics

The data are presented as means ± S.D. The differences between values before and after the 8-week endurance running program were tested using the paired samples t-test. For catecholamines only, the Wilcoxon signed-rank test was applied, since the Shapiro–Wilk test for normality revealed that their distributions were not normal. In addition, where significant differences were found, the effect size was evaluated using Cohen’s d for paired samples. Correlations of AO enzyme activities and the concentrations of GSH, MDA, A and NA with VO_2_max were tested using the Pearson product moment correlation coefficient. Statistical analyses were carried out using the Origin Pro 9 data analysis software (OriginLab Corporation, Northampton, MA, USA). A *p*-value at the level of 0.05 was considered significant.

## 3. Results

**Figure 2:**
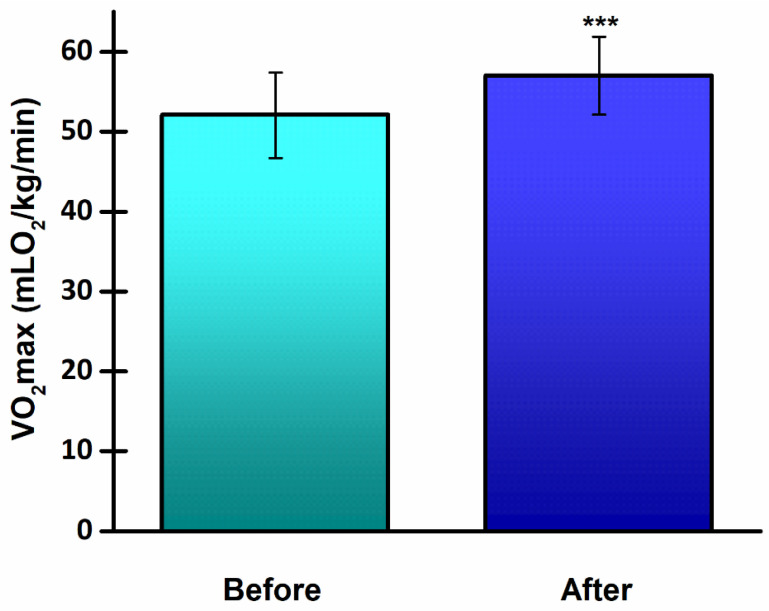
Maximal oxygen uptake (VO_2_max) in young, healthy athletes before and after the 8-week running program. *** *p* < 0.001, significantly different from before values.

The 8-week endurance training program resulted in a significant increase (*p* < 0.001) in VO_2_max (Figure 2). The effect size for VO_2_max was very large (d = 1.69).

**Figure 3:**
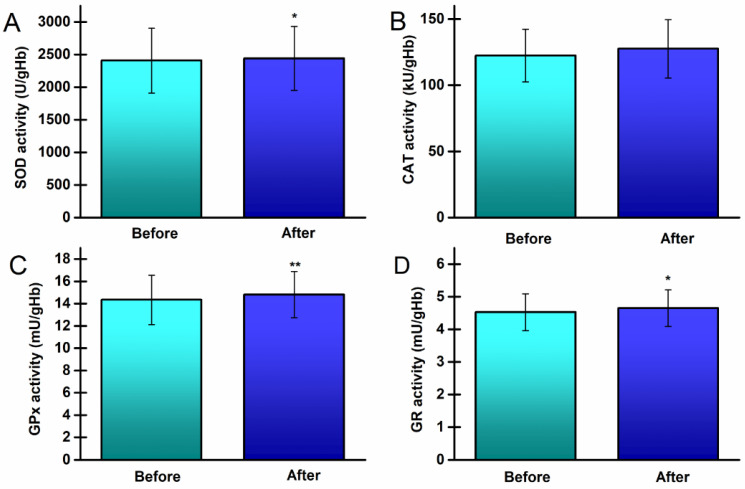
Antioxidant enzyme activity in peripheral blood of young, healthy athletes, before and after the 8-week running program: (**A**) superoxide dismutase (SOD), (**B**) catalase (CAT), (**C**) glutathione peroxidase (GPx), and (**D**) glutathione reductase (GR). * *p* < 0.05; ** *p* < 0.01, significantly different from before values.

The AO enzyme activities are presented in the Figure 3. 

The activities of SOD, GPx, and GR increased significantly (*p* = 0.014, *p* = 0.007, *p* = 0.015, respectively), with a small to medium effect size (d = 0.39, d = 0.43, and d = 0.39, respectively), while CAT activity remained unchanged. 

**Figure 4:**
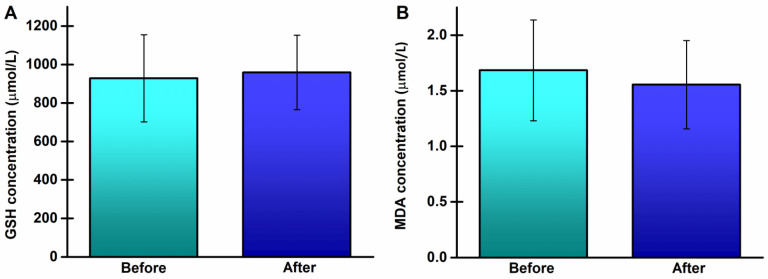
Concentrations of glutathione (GSH) and malondialdehyde (MDA) in peripheral blood of young, healthy athletes before and after the 8-week running program: (**A**) GSH; (**B**) MDA.

The concentrations of GSH and MDA also did not change significantly (Figure 4). 

**Figure 5:**
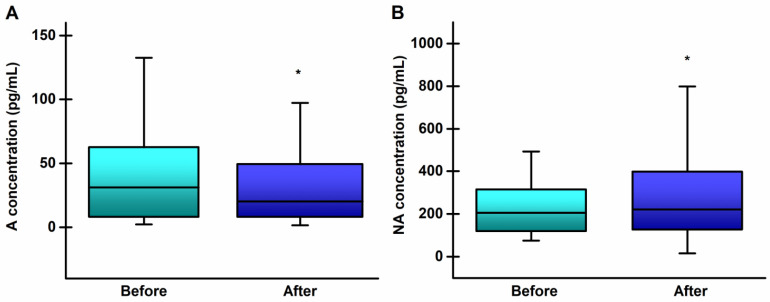
Concentrations of catecholamines in peripheral blood of young, healthy athletes, before and after the 8-week running program: (**A**) adrenaline (A); (**B**) noradrenaline (NA). Boxes represent values between the 25th and 75th percentile. Medians are given inside the boxes. Whiskers extend between min and max values. * *p* < 0.05, significantly different from before values.

The plasma concentrations of catecholamines are shown in the Figure 5. 

The training program led to a significant decrease in the plasma concentration of adrenaline (*p* = 0.031), while in contrast, the concentration of noradrenaline increased significantly (*p* = 0.015). The effect size for both hormones was small to medium (d = 0.32 and d = 0.33 for A and NA, respectively). 

**Figure 6:**
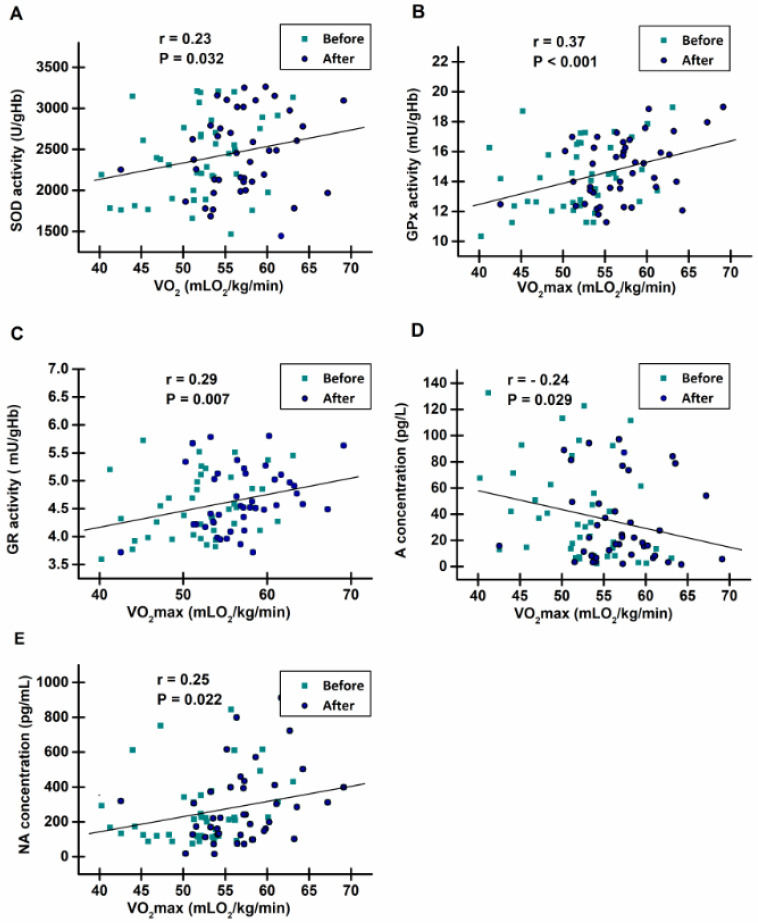
Data plot and coefficients of Pearson’s product moment correlation r between maximal oxygen uptake (VO_2_max) and: (**A**) superoxide dismutase (SOD) activity, (**B**) glutathione peroxidase (GPx) activity, (**C**) glutathione reductase (GR) activity, (**D**) concentration of adrenaline (A), and (**E**) concentration of noradrenaline (NA) in peripheral blood of young, healthy men before and after the 8-week running program.

Positive correlations were found between the activities of SOD, GPx, and GR (*p* = 0.032, *p* < 0.001, *p* < 0.007, respectively; Figure 6A–C) and VO_2_max. The plasma concentration of NA also positively correlated with VO_2_max (*p* = 0.022, Figure 6E), while a negative correlation was detected between the plasma concentration of A and VO_2_max (*p* = 0.029, Figure 6D).

The remaining analyzed parameters did not correlate with VO_2_max.

Summarized data are presented in Appendix A, specifically in Table A1.

## 4. Discussion

This study investigated the effect of the 8-week endurance running program on the activities of the AO enzymes and the concentrations of GSH, MDA, A, and NA. The training program was based on the standardized running protocol that led to a significant increase in VO_2_max and VO_2_ at VT1 and VT2 (results not presented here). The exercise program also resulted in increased activities of SOD, GPx, and GR. Moreover, these enzyme activities positively correlated with VO_2_max, although these correlations were weak, except in regards to GPx. 

Since exercise is followed by increased oxygen consumption, consequently, a higher level of ROS generation occurs. It has been estimated that aerobic exercise results in a 1–3-fold increase in the superoxide anion radical during muscle contraction [48]. The main sources of ROS in skeletal muscle are the mitochondrial respiratory chain, NADPH oxidase, and xanthine oxidase [49]. The elevated activities of AO enzymes found in this study may be considered a consequence of increased oxidative pressure. Our results are in agreement with those in the literature data [50,51,52,53]. Due to the invasiveness of obtaining human skeletal muscle biopsies, the majority of the findings regarding the influence of exercise on the AO defense system are based on blood analyses. Nevertheless, several studies demonstrate that endurance training leads to increased activities of the most important AO enzymes in human skeletal muscles [54,55,56]. 

Since CAT activity did not change significantly in the present study, it seems that hydrogen peroxide (H_2_O_2_) is predominantly eliminated by GPx. On the contrary, Knez et al. [51] confirmed that Ironman triathletes exhibited significantly higher resting activities for both GPx and CAT. This may reflect the kinetics of these enzymes, i.e., physiological levels of H_2_O_2_ are 1–10 up to 100 nM, while supraphysiological concentrations (>100 nM) indicate oxidative stress [57]. However, due to its high Michaelis–Menten constant (Km), CAT is the most effective at very high concentrations (mM) of H_2_O_2_ and will not respond to a moderate increase in substrate concentration. On the other hand, GPx, another H_2_O_2_ scavenger, provides better protection when H_2_O_2_ concentrations are lower [58,59]. We may presume that the AO defense system in Ironman triathletes must counteract much greater oxidative pressures provoked by ultra-endurance training when compared to the results for the subjects included in our study. 

The concentrations of GSH and MDA in the present study remained at the initial levels after the exercise program. MDA, as a final product of LPO, is a widely used biomarker of oxidative stress [60]. A key factor determining tissue susceptibility to injury by lipid peroxides is the GSH redox cycle [61,62]. Enzyme GPx uses GSH as a cofactor to detoxify lipid peroxides. GSH is then regenerated from its oxidized form (GSSG) by GR, with concomitant oxidation of NADPH. Apart from being an enzyme cofactor, GSH is one of the most important non-enzymatic antioxidants. It can directly react with free radicals and is very important in recycling chain-breaking antioxidants [63]. The results of the present study show that, despite increased consumption, GSH level is not seriously compromised, and GR plays an important role in this process. The effective GSH redox cycle successfully removes lipid peroxides, maintaining MDA at physiological levels. Our results are in agreement with the findings of Wagner et al. [64,65], who found that oxidative stress markers in the blood of triathletes and Ironman triathletes, although increased immediately after the race, returned to baseline after five days. Furthermore, lower resting plasma MDA levels were found in Ironman triathletes in comparison with those of the control subjects [51]. On the contrary, GSH decreased three days after an ultramarathon mountain race [66]. Strenuous aerobic exercise is commonly known to induce ROS overproduction due to enhanced metabolism, which is necessary to provide energy and to support continuous muscle contraction [67]. Whether and to what extent endurance exercise will produce oxidative damage depends on many factors, such as type, volume, intensity, and duration of exercise, as well as the ability of the AO defense system to detoxify free radicals. In this study, we confirmed that the applied endurance training program did not induce persisting oxidative damage. Some level of oxidative stress obviously existed, as expected, due to elevated oxygen consumption, but the AO defense system successfully counteracted increased oxidative pressure. On the other hand, since we have no insight into the processes that took place between the initial and final measurements, a potential phase of oxidative damage might have gone unnoticed. However, higher activities of SOD, GPx, and GR after the training program reflect exercise-induced adaptions to ROS overproduction, while the unchanged MDA concentration implies that the AO defense system is capable of maintaining prooxidant–antioxidant balance. Our results follow those of the published data. While acute intensive aerobic exercise may provoke oxidative muscle damage, regular aerobic exercise, on the contrary, enhances oxidative damage repair systems by increasing the activities of the AO enzymes SOD, GPx, and CAT [68,69,70]. 

Catecholamines A and NA are also known to be involved in the body’s response to stress induced by physical activity. They can act as protectors against oxidative stress, but under conditions of intense oxidative pressure, they can be vulnerable targets. [71,72] Exercise induces a catecholamine response, which is related to exercise intensity, as well as to the preceding training regimen [73]. The results of the present study show that the endurance training program significantly influenced the plasma concentrations of catecholamines. While the A concentration decreased after the training program, the NA concentration increased in comparison with the initial values. Physical activity may alter the hormonal response to a specific exercise workload. The secretion and the rate of release of hormones depend on their metabolic function and the requirements of given circumstances [74]. 

Literature data demonstrate that the plasma A response to aerobic exercise is rather small compared with the NA response [75]. On the contrary, brief high-intensity exercise significantly increases plasma A concentration. This type of exercise is predominantly anaerobic, and the energy for the working muscle is provided by glycolysis. A reduction in plasma glucose level induces the increased secretion of A, and the stimulation of glycogenolysis by A may be a safety backup mechanism, preventing hypoglycemia and the lack of fuel necessary for muscular contraction [76]. On the other hand, endurance training, which is essentially aerobic, requires different metabolic adjustments. The appropriate response of the sympathetic nervous system plays an important role in adjusting the physiological and metabolic requirements of the working muscles. An increased NA level is crucial for the cardiovascular stimulation, glycogenolysis, and lipolysis necessary for increasing the supply of oxygen and energy to the muscle during exercise [77,78]. The inversed response of the two catecholamines found in this study follows the findings of Greiwe et al. [75], who demonstrated that the mechanisms controlling the secretion of A by the adrenal glands and the NA release by the sympathetic nerve endings do not work in parallel. 

Our results confirm that an endurance training program induced adaptive alterations in the catecholamine system, which may be very important for the fine-tuning of neuroendocrine homeostasis. These adaptations may improve exercise capacity. Furthermore, since the same autoregulatory mechanisms are involved in the response to various stressors, the adaptations induced by physical stress could be beneficial under different stress conditions, including psychosocial environments. This can be very important in preventing stress-related diseases. 

The findings of this study must be considered in light of some limitations. The primary limitation to the generalization of our results is the fact that the study involved young healthy men. Different results could be obtained if the investigation included older subjects or people with health issues. Also, a study involving female subjects could provide different results, especially at the hormonal level, since it is known that there are differences between the sexes in the response to stress in general. Another limitation comes from the fact that we only possess initial and final values, so we can only speculate about the dynamics of the observed changes. On the other hand, this problem is not easily solved, since there are not many people who would be willing to give a blood sample several times over 8 weeks, or to wear a venous cannula that would allow several consecutive blood samplings before, during, and after individual training.

## 5. Conclusions

Our results indicate that despite an increase in ROS production, there is no persistent oxidative stress in response to an 8-week running program aimed at increasing aerobic endurance, probably due to exercise-induced protective alterations in the AO defense system. Furthermore, the applied training program induced adaptations at the hormonal level, making the organism more ready for a new challenge. Our results suggest that a long-term running program like the one applied in this study, leading to increased VO_2_max and increased oxygen consumption at VT1 and VT2, may be useful in preventing diseases caused by stress. An individual approach that considers the physical ability, previous experience, and state of health of each subject is necessary to avoid any undesirable effects of exercise.

## Data Availability

Data and materials are available from the corresponding author, upon reasonable and appropriate request.

## Appendix A

**Table A1:** Summarized data.

	**V** **O_2_ (mLO_2_/kg/min)**		**SOD (U/gHb)**		**CAT (kU7gHb)**		**GPx (mU7gHb)**			
	**Before**	**After**	**Before**	**After**	**Before**	**After**	**Before**	**After**		
1	54.18	57.28	3204.72	3250.73	104.89	95.95	13.07	12.28		
2	52.18	57.27	2206.32	2148.71	89.2	76.92	16.57	15.99		
3	40.25	42.55	2189.54	2251.95	148.28	126.18	10.33	12.46		
4	48.29	57.97	2309.77	2347.64	83.44	103.01	15.76	16.78		
5	52.08	54.06	3193.48	3157.34	96.6	106.86	12.36	12.16		
6	52.82	51.56	2283.69	2257.28	134.27	142.65	11.26	12.36		
7	44.22	53.65	1810.61	1968.82	107.33	113.06	12.36	13.26		
8	46.85	51.26	2394.22	2372.82	123.06	135.34	12.62	13.98		
9	60.11	67.24	1973.23	1967.83	102.19	120.27	17.86	17.96		
10	59.46	62.72	2889.85	2974.44	127.38	142.55	14.78	15.79		
11	61.28	60.92	2912.38	3151.12	112.48	126.62	13.37	14.24		
12	51.11	53.28	1657.06	1683.59	117.93	139.29	14.57	13.62		
13	63.12	69.13	3132.97	3093.86	85.64	105.31	18.95	18.99		
14	52.13	54.11	2678.13	2659.02	124.75	171.74	17.24	16.98		
15	51.69	59.82	3209.22	3261.8	112.18	118.7	16.47	17.57		
16	51.65	53.71	2215.13	2134.38	95.75	101.28	15.62	16.25		
17	41.25	50.28	1783.35	1863.98	118.59	124.89	16.26	16.02		
18	53.85	54.42	2704.05	2753.87	139.38	133.27	11.86	12.28		
19	56.35	56.85	2198.12	2158.8	129.88	129.8	14.21	13.98		
20	45.22	51.12	2607.9	2621.89	103.48	104.29	18.69	16.98		
21	48.68	54.25	1899.63	2127.2	113.96	155.88	12.00	11.78		
22	56.11	58.65	3198.99	3097.03	143.6	162.98	14.56	15.27		
23	52.69	57.45	1888.04	2002.26	122.86	138.79	17.26	16.25		
24	53.67	58.22	2163.68	2106.28	140.66	168.71	11.26	12.25		
25	50.08	53.28	2763.21	2788.38	138.19	121.05	12.32	13.38		
26	55.45	61.12	2447.19	2486.99	115.01	119.89	14.48	13.63		
27	42.58	53.56	1761.41	1765	115.69	104.62	14.18	15.18		
28	51.23	56.85	1997.63	1987.32	135.53	134.58	12.57	13.52		
29	47.28	56.35	2377.54	2455.02	158.57	187.47	14.24	15.63		
30	55.69	61.68	1465.99	1445.29	123.56	131.95	16.26	15.92		
31	53.48	59.63	2176.69	2193.9	168.24	117.04	14.49	15.22		
32	56.11	63.56	2552.8	2605.27	103.87	113.17	14.57	13.98		
33	43.95	55.21	3145.69	3103.67	129.64	143.77	11.24	11.27		
34	52.11	55.65	2650.9	2700.14	131.52	115.74	12.75	13.57		
35	59.16	64.28	2752.71	2779.79	144.21	133.58	12.65	12.06		
36	58.23	63.25	1754.7	1781.3	152.84	146.79	16.96	17.37		
37	53.36	57.18	2853.58	3016.34	114.38	118.8	14.06	15.73		
38	45.81	52.62	1765.61	1780.16	115.88	107.73	12.65	12.49		
39	56.18	60.26	2488.95	2485.17	132.03	118.08	17.32	18.85		
40	51.92	56.45	3070.23	3016.67	105.67	113.64	16.59	17.26		
41	51.28	57.22	1878.32	2104.95	154.03	152.26	12.06	16.62		
42	53.87	58.32	2565.56	2590.73	122.36	127.43	13.5	14.55		
	**GR (mU/gHb)**		**GSH (μmol/L)**		**MDA (μmol/L)**		**A (pg/mL)**		**NA (pg/mL)**	
	**Before**	**After**	**Before**	**After**	**Before**	**After**	**Before**	**After**	**Before**	**After**
1	3.96	4.11	622.35	676.69	1.65	2.17	2.2	2.1	120	433.3
2	5.26	5.22	514.69	697.63	2.06	1.85	22.2	76.9	226.7	73.3
3	3.59	3.72	1122.39	1065.99	1.82	1.14	67.4	15.8	293.3	320
4	4.69	4.52	635.27	677.54	1.36	1.44	40.6	73.5	126.7	186.7
5	4.22	3.98	811.2	761.41	1.65	1.38	96.3	3.6	246.7	160
6	3.85	4.22	618.34	753.8	1.42	2.35	30.2	3.4	200	173.3
7	3.92	4.25	720.59	645.69	1.58	1.31	71.3	8.5	226.7	312.1
8	4.26	4.22	1211.32	1070.23	2.52	1.22	50.7	49.3	120	306.7
9	5.37	4.49	711.15	1152.71	2.09	1.45	2.3	54.1	173.3	73.3
10	4.85	4.97	829.34	754.7	1.95	1.74	61.4	3.2	133.3	220
11	4.27	5.02	922.11	753.58	1.39	1.80	13.4	6.5	125	128
12	4.69	5.78	1386.25	865.61	1.69	1.31	13.6	22	75	170
13	5.45	5.63	1111.58	1388.95	2.25	1.08	6.2	5.6	166.7	18
14	5.11	5.13	958.36	750.9	1.28	1.83	5.8	6.6	120	127
15	4.98	5.27	857.95	1078.32	1.53	1.38	6.6	15.4	116	160
16	4.84	5.03	1056.89	672.82	1.74	1.37	6	3.2	120	16
17	5.2	5.34	695.11	851.95	1.34	1.15	132.5	89	430.2	398.6
18	3.82	3.95	878.35	748.71	2.68	2.41	56	48	156.8	222.6
19	4.52	3.86	1165.7	783.59	1.71	1.04	8.2	17	92.1	125.6
20	5.72	5.67	732.68	947.64	1.53	1.19	92.7	81.3	315.2	411.2
21	3.95	4.39	978.13	857.34	1.69	2.46	62.5	31.5	88.6	132.1
22	4.23	4.51	1209.22	957.28	1.03	1.00	14.1	22	611.2	571.3
23	5.22	5.13	615.13	1063.98	1.01	0.76	122.6	87.2	222.8	241.5
24	4.11	4.63	712.39	865.56	1.07	1.74	47.2	33.6	102	98.5
25	4.38	4.41	804.048	867.83	1.03	1.78	113	94.3	341.9	372.8
26	4.49	4.56	998.13	874.44	1.48	1.60	7.6	8.1	212.6	302.6
27	4.32	4.27	707.9	1268.82	1.80	2.02	12.8	8.2	615.3	722.1
28	4.03	4.55	999.63	950.73	2.26	1.95	84.5	97.2	311.6	459.2
29	4.55	4.72	1298.99	993.86	2.22	1.59	36.7	42.1	752	798.7
30	5.06	5.11	1088.04	1259.02	1.14	1.69	42.1	27.5	844.6	911.7
31	4.34	4.48	963.68	1161.8	2.64	1.79	33.6	18.2	113.8	148.3
32	4.58	4.77	863.68	1034.38	0.77	1.78	92.2	78.8	225.6	284.9
33	3.77	3.96	947.19	1151.12	1.69	1.29	42.1	37.2	611.6	615.2
34	3.96	4.09	989.54	1153.87	1.86	1.77	7.2	12.4	352.5	398.6
35	4.11	4.58	1206.32	958.8	2.24	1.81	2.8	1.6	492.9	502.8
36	4.86	4.91	1004.72	997.03	1.37	1.67	111.3	84.3	98.1	102.6
37	4.36	4.52	909.77	1127.2	1.79	1.30	22.8	24.2	88.6	111.7
38	3.98	4.17	1393.48	1121.89	1.98	1.08	14.6	11.3	376.4	392.8
39	5.51	5.8	1283.69	1102.26	1.45	1.21	18.5	16	210	198.5
40	5.52	5.37	810.61	1306.28	1.73	1.21	32.1	17.2	88.2	75
41	4.21	4.35	994.22	1088.38	1.14	1.39	17.5	22.6	215.6	241.7
42	3.94	3.72	629.35	986.99	2.05	1.81	8	9	117.3	98.2
